# Breakdown of Phospholipid Asymmetry Triggers ADAM17-Mediated Rescue Events in Cells Undergoing Apoptosis

**DOI:** 10.3390/membranes13080720

**Published:** 2023-08-05

**Authors:** Maria Sperrhacke, Sinje Leitzke, Björn Ahrens, Karina Reiss

**Affiliations:** Department of Dermatology, University of Kiel, 24105 Kiel, Germanybahrens@dermatology.uni-kiel.de (B.A.)

**Keywords:** scramblase, Xkr8, apoptosis, phosphatidylserine, EGFR ligands, Epiregulin

## Abstract

ADAM17, a prominent member of the “Disintegrin and Metalloproteinase” (ADAM) family, controls vital cellular functions through the cleavage of transmembrane substrates, including epidermal growth factor receptor (EGFR) ligands such as transforming growth factor (TGF)-alpha and Epiregulin (EREG). Several ADAM17 substrates are relevant to oncogenesis and tumor growth. We have presented evidence that surface exposure of phosphatidylserine (PS) is pivotal for ADAM17 to exert sheddase activity. The scramblase Xkr8 is instrumental for calcium-independent exposure of PS in apoptotic cells. Xkr8 can be dually activated by caspase-3 and by kinases. In this investigation, we examined whether Xkr8 would modulate ADAM17 activity under apoptotic and non-apoptotic conditions. Overexpression of Xkr8 in HEK293T cells led to significantly increased caspase-dependent as well as PMA-induced release of EREG and TGF-alpha. Conversely, siRNA-mediated downregulation of Xkr8 in colorectal Caco-2 cancer cells led to decreased PS externalization upon induction of apoptosis, which was accompanied by reduced shedding of endogenously expressed EREG and reduced cell survival. We conclude that Xkr8 shares with conventional scramblases the propensity to upmodulate the ADAM-sheddase function. Liberation of growth factors could serve a rescue function in cells on the pathway to apoptotic death.

## 1. Introduction

Lipid asymmetry in eukaryotic cell membranes is upheld by ATP-dependent flippases that actively transfer phosphatidylserine (PS) and phosphatidylethanolamine (PE) from the outer to the inner leaflet [1,2,3]. The transient breakdown of membrane asymmetry occurs continuously as a dynamic event that serves to coordinate many processes in the cells [4,5,6,7]. Permanent surface exposure of PS is a key signal for apoptotic cell clearance.

PS externalization occurs through the action of scramblases, the functional counterparts of flippases, that translocate phospholipids bidirectionally along their concentration gradients. Three major scramblase families have been identified that differ in their activation mechanisms. The first comprises members of Ca^2+^-activated proteins belonging to the transmembrane protein (TMEM)16 (also called anoctamin) family [8]. The second group includes Class A (rhodopsin-like) G protein-coupled receptors that are constitutively active [9]. The third type is a member of the Xkr family, the most prominent of which is Xkr8. This ubiquitously expressed scramblase is activated by caspase-mediated cleavage in its C-terminal region [10,11], which induces its dimerization into the active scramblase that is pivotal to PS exposure in cells undergoing apoptosis. In parallel, caspases inactivate flippases, thus counteracting the restoration of membrane asymmetry [12].

Xkr8-deficient cells undergoing apoptosis do not expose PS [11]. This finding renders it apparent that the externalization of PS subserves functions that are unrelated to the death pathway itself. It is known that surface exposure of PS is a key signal for apoptotic cell clearance. In this study, it is shown that PS also activates cell-bound metalloproteinases. The resulting release of growth factors may play a relevant role in countering the apoptotic cascade. 

ADAMs are transmembrane multidomain proteins implicated in a multitude of biological processes. ADAM17, originally identified as the TNF-alpha releasing enzyme [13,14], is involved in the shedding of many cell surface proteins, including the epidermal growth factor receptor (EGFR) ligands transforming growth factor-alpha (TGF-alpha), Epiregulin (EREG), or Amphiregulin (AREG) [15,16]. EGFR ligands play important roles in tumor development and metastasis [17]. Constitutive expression of EREG is low in normal tissues but elevated in various cancer types, such as non-small cell lung cancer, breast cancer, gastric cancer, head and neck cancer, ovarian cancer, colorectal cancer, brain cancer, bladder cancer, and is thought to promote tumor progression [17,18]. Upregulation of EREG in colorectal cancer tissues correlates with depth of tumor invasion, distant metastases, and poor prognosis [19].

Functional upregulation of ADAM17 in living cells is mediated via diverse pathways, including cytosolic Ca^2+^ elevation and activation of protein kinase C (PKC) and tyrosine kinases [20,21,22,23,24]. We have reported that these pathways culminate in the externalization of PS, which in the case of Ca^2+^-dependent stimuli is mediated by anoctamins [25,26,27,28]. The putative scramblase responsible for PKC-induced PS exposure has remained unknown. Externalized PS interacts with a cationic motif located in the membrane-proximal domain of ADAM17. The protease is thus guided to the membrane surface, where it cleaves its target [29].

The objective of this study was to examine whether Xkr8 expression and activation would also modulate ADAM17-mediated shedding. We show that this is the case. Shedding of classical ADAM17 substrates TGF-alpha and EREG was significantly enhanced in Xkr8-transfected HEK293T cells upon induction of apoptosis. siRNA-mediated downregulation of *Xkr8* led to diminished PS exposure and reduced release of EREG in a colorectal tumor cell line. This was accompanied by decreased ATP levels compared with mock-transfected cells. The collective results tie in with the concept that enhanced expression of ADAM-liberated growth factors may increase the resilience of tumors to apoptotic stimuli.

## 2. Materials and Methods

### 2.1. Reagents and Antibodies

Annexin V-Fluor 488 was from Thermo Fisher Scientific (Waltham, MA, USA). O-phospho-L-serine (OPS), Phorbol 12-myristate-13-acetate (PMA), and cycloheximide (CHX) were from Sigma Aldrich (St. Louis, MO, USA). The hydroxamate-based ADAM17/ADAM10 inhibitor GW280264 (GW) was purchased from Aeobious (Gloucester, MA, USA). Marimastat and the ADAM10 inhibitor GI254023X (GI) were purchased from Tocris Bioscience (Bristol, UK). The neutralizing ADAM17 antibody D1 (A12) was purchased from AdipoGene Life Sciences (Fuellinsdorf, Switzerland, AG-27B-6000PF-C100). Antibodies against EGFR, AKT, full-length caspase-3, cleaved caspase-3, and actin were from Cell Signaling Technology (Danvers, MA, USA). Anti-mCherry antibodies were from Novus Biologicals/Bio-Techne (Wiesbaden, Germany). Anti-ADAM17 antibodies used for Western blotting were from Merck Millipore (Darmstadt, Germany; AB19027) and from Cell Signaling Technology (3976S). Recombinant human killer TRAIL (kTRAIL), which utilizes a linker peptide to increase cross-linking to form a more stable oligomer, was from ENZO Life Sciences (Lörrach, Germany); pan-caspase inhibitor zVAD-fmk (ZVAD) was from BACHEM (Bubendorf, Switzerland). The live dead dye 7-Aminoactinomycin D (7-AAD) was from BioLegend (San Diego, CA, USA). BAPTA-AM was from MedChemExpress (Monmouth Junction, NJ, USA).

### 2.2. Cell Culture

HEK293T cells as well as Caco-2 cells were from Sigma Aldrich (St. Louis, MO, USA). HEK293T cells and ADAM10/ADAM17 double-deficient HEK293 cells [30] were grown in DMEM (High glucose; Thermo Fisher Scientific) supplemented with 10% fetal calf serum (FCS) and 1% penicillin/streptomycin (Pen/Strep). Caco-2 cells were cultured in RPMI (Thermo Fisher Scientific) with 10% FCS and 1% Pen/Strep.

### 2.3. Expression Vectors

Human Xkr8-mCherry expression vector (pReceiver (EX-V1531-M55) was from GeneCopoeia (Biocat, Heidelberg, Germany). Alkaline phosphatase (AP)-tagged Epiregulin and TGF-alpha were kindly provided by Dr. Carl P. Blobel (Hospital for Special Surgery, New York, NY, USA). The constructs are used to express truncated ADAM substrates, where most of the natural ectodomain is replaced by AP as a readout for shedding activity, as described in ref. [15]. Human AP is fused to the membrane-proximal EGF repeat, followed by the juxtamembrane, transmembrane, and cytoplasmic C-terminal regions of the growth factors (EREG 110 amino acids, TGF-alpha 115 amino acids).

### 2.4. Biotinylation

Cell surface proteins were isolated with a Pierce Cell Surface Protein Isolation Kit (Thermo Fisher Scientific). In brief, cells were seeded in 10 cm dishes (1.5 × 10^6^) and transfected the next day (6 µg plasmid with 12 µL Turbofect in 100 µL DMEM). 48 h after transfection, cells were washed twice with PBS on ice. After incubation with a 10 mL Sulfo-NHS-SS-Biotin solution for 30 min at 4 °C, cells were washed once with PBS, and then a quenching solution was added. Cells were washed twice and lysed in lysis buffer (5 mM Tris-HCl [pH 7.5], 1 mM EGTA, 250 mM saccharose, and 1% Triton X-100) supplemented with cOmplete inhibitor cocktail (Roche Applied Science, Penzberg, Germany) and 10 mM 1,10-phenanthroline monohydrate and centrifuged. Supernatants were incubated with NeutrAvidin Agarose beads for 2 h to capture labeled proteins. After three washing steps, proteins were eluted with 1× SDS sample buffer at 95 °C for 10 min. The analysis of the cell surface fraction and cell lysates was performed via automated immunoblot. Positive EGFR staining (Cell Signaling Technology, #4267P) was indicative of proteins located on the cell surface. Akt staining (Cell Signaling Technology, #2920S) served as a control for cytosolic proteins, and mCherry staining (Novus Biologicals/Bio-Techne, #NBP2-25157) for the detection of Xkr8. 

### 2.5. Transfection and AP-Substrate Shedding Assay in HEK293T Cells 

HEK293T cells were transfected using Turbofect Transfection Reagent (Thermo Fisher Scientific) according to the manufacturer’s instructions. Transfection efficiency was always controlled by visual inspection. For induction of apoptosis, 24 h after transfection cells were treated with kTRAIL (50 ng/mL) overnight. CHX (5 µg/mL), which inhibits protein synthesis, was used for cell sensitization. Control cells were treated with CHX only. For PMA stimulation, cells were treated with PMA (100 ng/mL, 30 min) 48 h after transfection in the absence or presence of the indicated inhibitors. Before treatment, the cell medium was changed to a fresh medium. Assays were analyzed as described [25]. 

### 2.6. Annexin V Staining

Annexin staining of HEK293T cells was performed as described [25]. 

### 2.7. Image Analysis and Image Statistics

Image analysis was carried out as described [27]. Results were tested by one-way analysis of variance and the Holm-Sìdàk multiple comparison post hoc test.

### 2.8. Determination of Caspase Activity in HEK293T Cells

Caspase-3 activity was determined using Ac-DEVD-AMC (ENZO), a fluorogenic caspase 3 substrate. HEK293T cells were seeded in 48-well plates (35.000 cells/well) and transfected with a mock vector or Xkr8. 24 h after transfection, cells were treated with kTRAIL (50 ng/mL) and CHX (5 µg/mL) with or without ZVAD (50 µM) overnight. Cells were harvested by lysing cells with RIPA buffer. 50 µL of cell lysate was added to 150 µL of assay buffer containing 50 µM Ac-DEVD-AMC, 100 mM HEPES pH 7,4, 10% Sucrose, 0.1% CHAPS, 100 mM NaCl, and 5 mM DTT. After 1 h incubation at 37 °C, fluorescence was monitored using an Infinite^®^ 200 PRO MPLEX (TECAN, Männedorf, canton of Zürich, Switzerland) every 10 min at 37 °C with excitation at 355 nm and emission at 460 nm over 3 h. Caspase activity was expressed as a slope in fluorescence units per time.

### 2.9. siRNA Transfection and Induction of Apoptosis

Caco-2 cells and HEK cells were transfected with Xkr8 siRNA and control siRNA using Lipofectamine RNAiMAX (Invitrogen) according to the manufacturer’s instructions. *Xkr8 Silencer*^®^ Select (siRNA ID: s30202) and *Silencer*^®^ Select Negative Control #2 siRNA were from Thermo Fisher Scientific. HEK cells were transfected with AP-tagged EREG or TGF-alpha 24 h after siRNA transfection. 48 h after siRNA transfection, cells were either treated with CHX (5 µg/mL) only (mock) or treated with kTRAIL (100 ng/mL for Caco-2; 50 ng/mL for HEK) and CHX in the absence or presence of ZVAD (50 µM) overnight. 72 h after siRNA transfection, *Xkr8* expression in Caco-2 cells was determined by qPCR, and the release of soluble EREG as well as ATP content was monitored by ELISA and cell viability assays, respectively. HEK cells were analyzed for AP-substrate shedding and for *Xkr8* expression.

### 2.10. Epiregulin ELISA

Epiregulin ELISA (R&D, Minneapolis, MN, USA) was performed according to the manufacturer′s instructions. Caco-2 cells were seeded in 6-well plates. 48 h after transfection of mock siRNA or Xkr8 siRNA, cells were treated with CHX with or without kTRAIL in the absence or presence of ZVAD overnight. Furthermore, the medium was exchanged, harvested after 1 h, and analyzed for soluble EREG via ELSIA in duplicates. Cells were used for FACS analysis of PS exposure in parallel. 

### 2.11. Immunoblot Analysis

The analysis of biotinylation experiments was completed via Automated Western and evaluated as stated before [25]. The following primary antibodies were used: mCherry (1:30), EGFR (1:30), AKT (1:20), actin (1:10), caspase-3 (1:15), and cleaved caspase-3 (1:40) followed by the respective secondary antibodies (Bio-Techne). The analysis of ADAM17 protein amounts was performed by classical immunoblot as described in ref. [31], since these antibodies do not work in Automated Western.

### 2.12. Real-Time PCR of Xkr8 Expression

RT-PCR auf *Xkr8* expression was basically performed as described [27] with the following modifications. Total RNA was extracted with the NucleoSpin RNA Kit (MACHERY-NAGEL, Düren, Germany) RT-qPCR was performed using a QuantStudio 3 instrument (ThermoFisher Scientific). The following primers were applied: hHPRT1 5′-TGGCGTCGTGATTAGTGATG-3′ (fwd) 5′-TCTCGAGCAAGACGTTCAGT-3′ (rev), hXkr8 5′-GCAGCTGGGTTACCTGTACA-3′ (fwd) 5′-TGATGGCCAGCACCAGCGTGA-3′ (rev) (all purchased from Sigma Aldrich).

### 2.13. Flow Cytometric Analysis (FACS) of PS Exposure

Caco-2 and HEK cells were seeded in 6-well plates (Caco-2 500.000/well; HEK 250,000/well) and transfected with mock siRNA or Xkr8 siRNA. 48 h after transfection, cells were either treated with CHX (mock control) or treated with kTRAIL/CHX in the absence or presence of ZVAD overnight. Cells were incubated with accutase (Merck Millipore, Darmstadt, Germany) for detachment, harvested, and stained with Annexin V-Fluor 488 (5 µL) and 7-AAD (2.5 µL) in 100 µL of ABB at room temperature for 15 min. PS exposure was analyzed using a CytoFlex flow cytometer (Beckman Coulter, Brea, CA, USA) and FlowJo 10.8.1 software.

### 2.14. Cell Viability Assay

A luminometric ATP assay (CellTiter-Glo, Promega, Walldorf, Germany) was performed to determine cell viability. Caco-2 cells were seeded in 96 well plates (20.000/well) and transfected with a mock siRNA or Xkr8 siRNA. After 24 h, kTRAIL (100 ng/mL) and CHX were applied in the absence or presence of ZVAD (50 µM) overnight. The medium was exchanged, and an equal volume of CellTiter-Glo^®^ Reagent was added. Luminescence was monitored in duplicate using an Infinite^®^ 200 PRO MPLEX (TECAN).

### 2.15. Statistics

Data represent means ± standard error of the mean (SEM) obtained from at least three independent experiments. Statistics were generated using a one-way analysis of variance and the Holm-Sídák multiple comparison post hoc test if not otherwise specified. 

## 3. Results

### 3.1. HEK293T Cells Overexpressing Xkr8 Display Increased Shedding of TGF-Alpha and EREG upon Induction of Apoptosis

**Figure 1:**
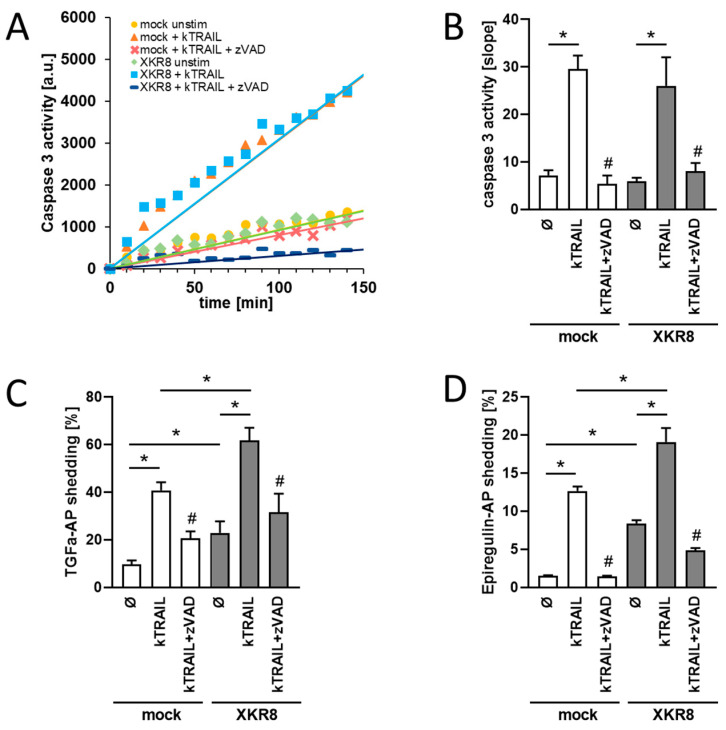
Caspase-3 activation induces increased shedding of ADAM17 substrates in Xkr8 overexpressing cells. (**A**) Caspase-3 is similarily activated in HEK293T cells transfected with Xkr8 or mock vector upon stimulation with kTRAIL (50 ng/mL) and CHX (5 µg/mL) and reduced in the presence of pan-caspase inhibitor ZVAD (50 µM). Caspase-3 activity of one representative experiment over time. (**B**) Quantification of caspase-3 activity experiments (*n* = 4; */# *p* < 0.05; ±SEM). (**C**,**D**) HEK293T cells were co-transfected with Xkr8 or mock vector and AP-tagged ADAM17 substrates TGF-alpha (**C**) or epiregulin (**D**), respectively. Cells were stimulated CHX (5 µg/mL) alone (mock) or with kTRAIL (50 ng/mL) and CHX (5 µg/mL) overnight in the presence of absence of caspase inhibitor ZVAD (50 µM). Constitutive and apoptosis-induced shedding was significantly increased upon overexpression of Xkr8. * Significant increase compared with indicated control, # significant decrease compared with respective kTRAIL-treated cells. (**C**,**D**) *n* = 3; */# *p* < 0.05; ±SEM).

Xkr8 has been shown to mediate PS exposure in response to apoptotic stimuli [11,31]. We first questioned whether this would result in the functional activation of ADAM17. Biotinylation experiments were performed to ensure that transfected Xkr8 would be localized on the cell surface upon transfection in HEK cells. As shown in Supplementary Figure S1, this was the case. Furthermore, experiments were conducted to examine whether overexpression of Xkr8 would lead to enhanced ADAM17 sheddase activity upon induction of apoptosis. HEK cells were treated with recombinant cross-linked Tumor necrosis factor (TNF)-related apoptosis-inducing ligand (kTRAIL) in the presence or absence of the pan-caspase inhibitor zVAD-fmk (ZVAD). Caspase-3 activation was monitored with the caspase substrate DEVD-AMC assay. As shown in Figure 1A,B, caspase-3 was activated to a comparable extent in mock- and Xkr8-transfected cells (Figure 1A,B). The caspase inhibitor ZVAD completely abolished caspase-3 induction. For analysis of sheddase activation, HEK293T cells were transfected with alkaline-phosphatase (AP)-tagged TGF-alpha or EREG to provide two common ADAM17 targets [15,22]. Cells were stimulated overnight with kTRAIL in the presence or absence of ZVAD. Induction of apoptosis led to increased shedding of TGF-alpha and EREG (Figure 1C,D). This was significantly enhanced in Xkr8-transfected cells compared with mock-transfected cells. The caspase inhibitor ZVAD reduced sheddase activation to constitutive shedding levels. Of note, Xkr8 overexpression has already increased constitutive sheddase activity in the absence of any stimulus. Altered *Xkr8* expression did not apparently affect protein levels of ADAM17 (Supplementary Figure S2). 

### 3.2. Xkr8 Overexpression Leads to Enhanced PMA-Induced Shedding of TGF-Alpha and EREG

**Figure 2:**
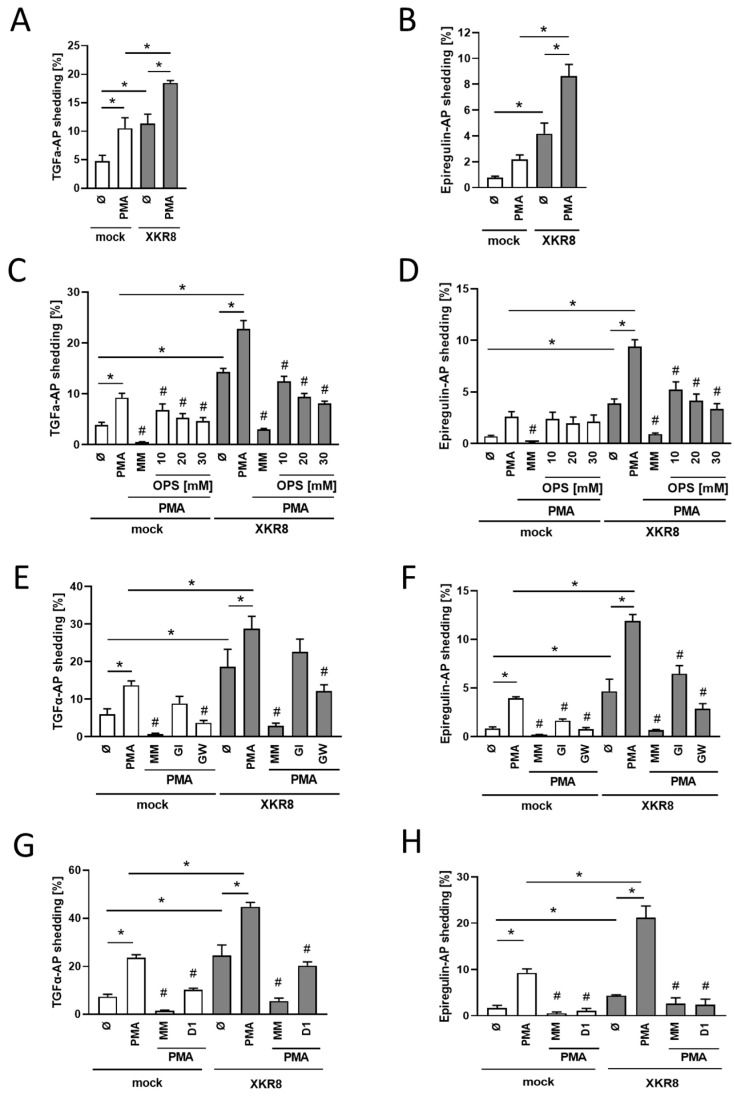
PMA-stimulation leads to increased shedding of TGF-alpha and EREG in Xkr8 overexpressing cells. HEK293T cells were co-transfected with mock vector or Xkr8 and (**A**) AP-tagged TGF-alpha or (**B**) EREG, respectively. Cells were stimulated with PMA (100 ng/mL) for 30 min and analyzed for substrate shedding. (**C**,**D**) HEK293T cells were analyzed for TGF-alpha (**C**) or EREG (**D**) shedding after 30 min in the presence or absence of broad-spectrum metalloprotease inhibitor marimastat (MM, 10 µM) or OPS. (**E**,**F**) Cells were stimulated with PMA (100 ng/mL) for 30 min in the presence or absence of marimastat (MM, 10 µM), ADAM10 inhibitor GI (3 µM), or the ADAM17/ADAM10 inhibitor GW (3 µM) and analyzed for substrate shedding. (**G**,**H**) Cells were stimulated with PMA (100 ng/mL) for 30 min in the presence or absence of marimastat (MM, 10 µM)) or the ADAM17 inhibitory antibody D1 (200 nM) and analyzed for substrate shedding. * significant increase compared with indicated control, # significant decrease compared with corresponding stimulated cells. (**A**–**D**,**E**,**G**): *n* ≥ 4; ±SEM. (**F**,**H**): *n* = 3 ± SEM; */# *p* < 0.05).

We previously found that PKC-activation by PMA also promotes PS-externalization, which triggers ADAM17 sheddase function [28]. Anoctamin-scramblases could not have been responsible because they are not subject to PKC-activation. At that time, the open question remained whether a PKC-activatable scramblase might exist. Subsequently, it was discovered that murine Xkr8 can be activated by phosphorylation [32]. Experiments were accordingly undertaken to determine whether Xkr8-transfection would also enhance PMA-induced shedding. This was indeed the case (Figure 2A,B). The soluble PS-headgroup phosphorylserine (OPS, O-phospho-l-serine) was employed as a competitive inhibitor to ensure that shedding was specifically mediated by PS. OPS dose-dependently decreased TGF-alpha and EREG shedding (Figure 2C,D).

Previous experiments had shown that anoctamin overexpression and activation could shift the substrate specificity of the ADAMs. In particular, TGF-alpha release was then no longer effected by ADAM17 [25,27]. Analogous experiments were conducted to discern whether similar observations would be made in Xkr8-transfected PMA-stimulated cells. The preferential ADAM10 inhibitor GI254023X (GI) and GW280264X (GW), a dual inhibitor of ADAM10/17 [33,34], were employed to differentiate between the ADAMs involved. As expected, PMA-induced shedding of TGF-alpha was only slightly reduced by GI but significantly inhibited by GW in mock-transfected cells (Figure 2E). A similar finding was made for TGF-alpha release in Xkr8-transfected cells. This finding indicates that, in contrast to anoctamin overexpression, ADAM17 remains the major convertase of TGF-alpha in cells overexpressing Xkr8. In a similar vein, Xkr8 overexpression provoked no change in the ADAMs involved in EREG-cleavage. As in mock-transfected cells, shedding was partially reduced by the ADAM10 inhibitor GI and substantially inhibited by the dual ADAM10/17 inhibitor GW (Figure 2F). Essentially complete inhibition of substrate release was observed when the broad-spectrum metalloprotease inhibitor marimastat was applied. The key role of ADAM17 was confirmed through the use of the ADAM17-specific inhibitory antibody D1 [35], which significantly suppressed EREG release to almost the same extent as marimastat. Application of D1 also led to a significant reduction in the shedding of TGF-alpha in both mock-transfected and Xkr8-transfected cells (Figure 2G,H). It is known from TGF-alpha release that ADAM10 can take over upon the loss of ADAM17 function [24]. Indeed, this seems to be the case since ADAM10/ADAM17 double-deficient HEK cells showed neither TGF-alpha nor EREG shedding under mock or Xkr8-transfected conditions (Supplementary Figure S3).

### 3.3. Increased Constitutive Substrate Cleavage in Xkr8-Overexpressing Cells 

**Figure 3:**
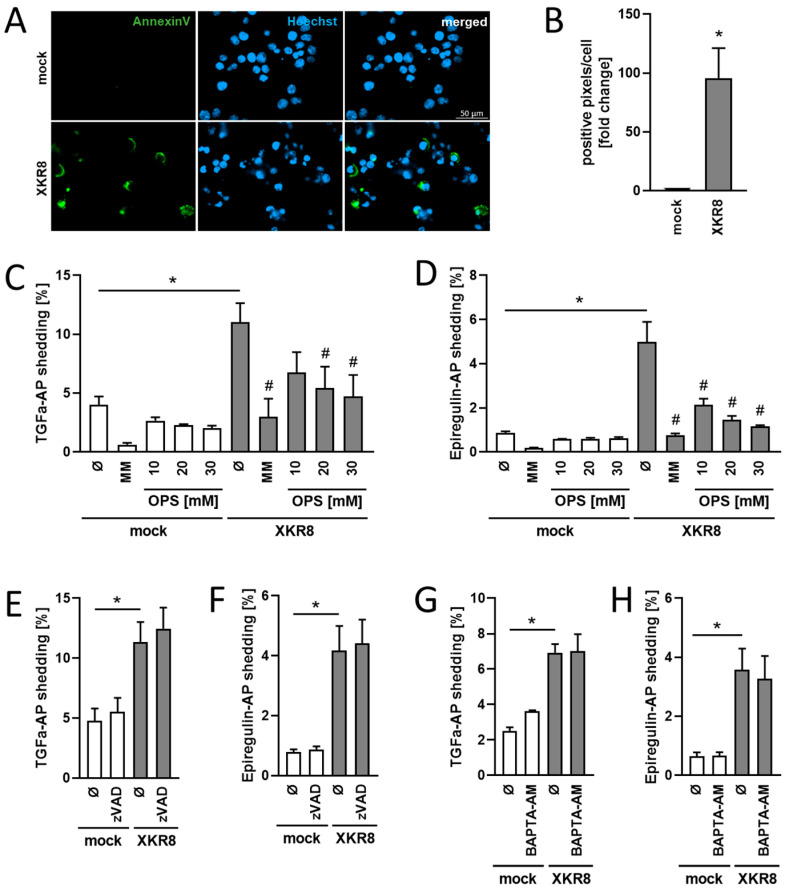
Increased constitutive PS exposure and substrate cleavage in Xkr8-overexpressing cells. HEK293T cells were co-transfected with mock vector or Xkr8 and stained with Annexin V-Fluor 488 (green) and Hoechst (blue) 48 h after transfection. (**A**) Representative images of four independent experiments. (**B**) The fluorescence was quantified for statistical analysis (*n* = 4; * *p* < 0.05; ±SEM). * significant increase compared with mock-transfeced controls. (**C**,**D**) Constitutive shedding of AP-tagged TGF-alpha (**C**) or EREG (**D**) was analyses in the presence of marimastat (MM, 10 µM) and OPS for 30 min (*n* = 3; */# *p* < 0.05; ±SEM). (**E**,**F**) Elevated constitutive shedding in Xkr8 overexpressing cells is caspase-independent. Mock and Xkr8-transfected HEK293T cells were incubated with or without pan-caspase inhibitor ZVAD (50 µM) and analyzed for shedding of TGF-alpha (**E**) or EREG (**F**) (*n* ≥ 4; * *p* < 0.05; ±SEM). (**G**,**H**) Elevated constitutive shedding in Xkr8 overexpressing cells is independent of intracellular calcium. Calcium chelator BAPTA-AM (25 µM) was applied before analysis of TGF-alpha (**G**) and EREG (**H**) shedding (*n* = 3; * *p* < 0.05; ±SEM). * significant increase compared with respective controls, # significant decrease compared with corresponding control cells.

Sakuragi et al. [32] have shown that overexpression of murine Xkr8 leads to spontaneous, constitutive PS-scrambling in mouse Ba/F3 B cells. This constitutive scrambling activity was Ca^2+^-dependent and could be inhibited with the calcium chelator BAPTA-AM. In our experiments, we also found that overexpression of human Xkr8 in HEK cells led to enhanced constitutive PS exposure (Figure 3A,B) and to increased constitutive shedding of TGF-alpha and EREG that was dose-dependently reduced in the presence of OPS (Figure 3C,D). Preincubation with pan-caspase inhibitor ZVAD or calcium chelator BAPTA-AM did not decrease EREG or TGF-alpha release, indicating that the constitutive shedding activity invoked by overexpression of human Xkr8 was independent of both caspase activation (Figure 3E,F) and calcium (Figure 3G,H).

### 3.4. Downregulation of Xkr8 Diminishes PS-Externalization, Growth Factor Release and Cellular Resilience to Apoptosis

**Figure 4:**
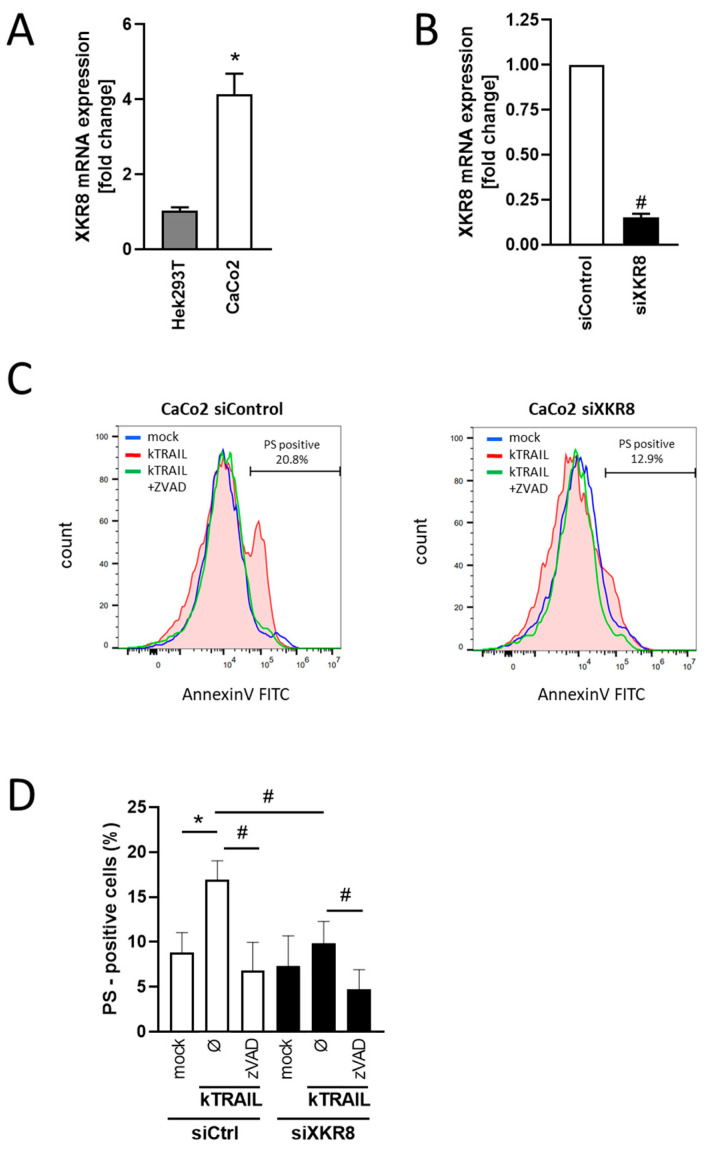
Decreased expression of *Xkr8* in Caco-2 cells leads to decreased kTRAIL-induced PS exposure. (**A**) *Xkr8* expression in HEK and Caco-2 cells was analyzed via qRT-PCR. * significant difference, (*n* = 3; * *p* < 0.05; ±SEM; unpaired *t*-test). (**B**) Caco-2 cells were either transfected with control siRNA or with Xkr8 siRNA. After 72 h, cells were analyzed for *Xkr8* expression by qRT-PCR. # significant decrease (*n* = 3; # *p* < 0.05; ±SEM; unpaired *t*-test). (**C**) Caspase-dependent PS exposure is decreased in Xkr8 siRNA transfected cells. Mock transfected and Xkr8 siRNA transfected Caco-2 cells were stimulated with CHX (5 µg/mL) alone (mock) or with kTRAIL (100 ng/mL) and CHX in the absence or presence of ZVAD (50 µM), stained for 15 min with Annexin V-488 and 7-AAD for exclusion of dead cells and analysed via FACS. The Annexin V-staining profiles of the FITC-positive and 7-AAD-negative population of one representative experiment are shown. (**D**) Quantifaction of viable PS-positive cells of three independent experiments (*n* = 3; */# *p* < 0.05; ±SEM). * significant increase compared with respective control, # significant decrease compared with corresponding cells.

siRNA-mediated downregulation of Xkr8 reportedly leads to colorectal tumor reduction [36], and the question followed whether modulation of ADAM sheddase activity might play a role in this context. The next experiments were conducted with the colorectal tumor cell line Caco-2. In contrast to HEK cells, these cells endogenously express the EGFR ligand EREG, whose cleavage by ADAM17 can be directly assessed via ELISA. Expression of Xkr8 is also manifold higher than in HEK cells (Figure 4A) and thus amenable to down-regulation. Caco-2 cells were either mock-transfected or transfected with Xkr8 siRNA. RT-PCR analyses conducted after 48 h showed that *Xkr8* expression was reduced by 80% (Figure 4B). Cells were exposed to kTRAIL, which induces apoptosis in Caco-2 cells in the presence of the protein biosynthesis inhibitor CHX (Supplementary Figure S4) [37]. After overnight incubation, Annexin V FITC staining profiles of the viable (7-AAD-negative) cell population demonstrated that downregulation of *Xkr8* significantly reduced the numbers of cells with exposed PS (Figure 4C,D).

**Figure 5:**
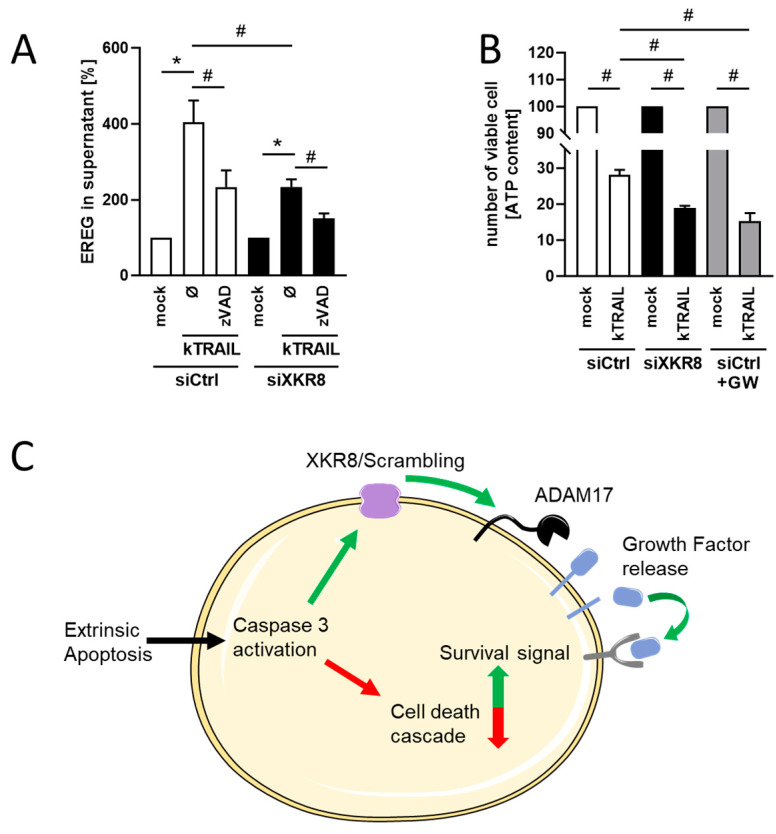
Decreased expression of *Xkr8* leads to decreased release of soluble EREG and decreased cellular ATP content. (**A**) Caco-2 cells were either transfected with control siRNA (siCtr) or with Xkr8 siRNA (siXkr8). After 48 h, cells were stimulated with CHX (5 µg/mL, mock) or with kTRAIL (100 ng/mL) and CHX in the absence or presence of ZVAD (50 µM) and analyzed for release of soluble EREG via ELISA. (**B**) Caco-2 cells were either transfected with control siRNA or with Xkr8 siRNA and either mock stimulated or stimulated with kTRAIL/CHX. Mock-transfected cells were additionally incubated in the presence of ADAM10/ADAM17 inhibitor GW (3 µM). After overnight incubation, ATP was measured using a luminometric ATP cell viability assay. The kTRAIL-treated cells are compared with the respective non-treated controls, which were set to 100 percent (*n* = 3; * *p* < 0.05; ±SEM). * significant increase compared with respective control, # significant decrease compared with corresponding cells. (**C**) Proposed model of Xkr8-dependent ADAM activation. Caspase activation induces phospholipid scrambling via Xkr8 or related scramblases. PS exposure activates ADAM17 sheddase function and release of growth factors that might counteract the death cascade.

Decreased PS-externalization would be expected to diminish ADAM sheddase activity. Indeed, release of EREG was significantly reduced in Xkr8 siRNA-treated cells following induction of apoptosis (Figure 5A). Among its numerous functions, EREG has been reported to confer resistance to apoptosis [18,38]. Cellular ATP was accordingly assessed to discern whether reduced EREG release was accompanied by an augmentation of cell death. As shown in Figure 5B, kTRAIL treatment reduced ATP to 30% of starting levels in siControl cells but to less than 20% in siXkr8-transfected cells. Application of the ADAM10/ADAM17 inhibitor GW caused an effect very similar to siRNA-treatment (Figure 5B, grey columns). Neither inhibitor treatment nor Xkr8 siRNA significantly changed the ATP content compared with mock-transfected cells under non-stimulated conditions (Supplementary Figure S5). 

The results identified Xkr8 as the major scramblase responsible for PS-externalization during apoptosis of Caco-2 cells. The question followed whether knockdown in cells equipped with low Xkr8 levels would have any similar effects, and experiments were accordingly conducted with HEK-cells. Xkr8 siRNA did cause further reduction of *Xkr8*, but in this case, no significant effect on apoptosis-triggered PS-externalization or shedding of EREG and TGF-alpha was observed (Supplementary Figure S6). These results suggest that one or several other caspase-activatable members of the Xkr-family may functionally replace Xkr8 in HEK cells.

## 4. Discussion

The significance of membrane asymmetry and the traffic of PS to the outer leaflet is currently a subject of intense interest. It has become clear that a multitude of cell biological events are triggered by surface-exposed PS and are highly relevant in apoptosis, infections, and oncogenesis. Blockade of PS externalization is discussed as part of anti-tumor immunotherapy [39]. In a previous contribution to this expanding field, we reported that PS-exposure leads to immediate upregulation of ADAM-sheddase activity [25,27,28,40]. This, in turn, would trigger a plethora of events relevant to cell growth and survival. Externalization of PS was observed to occur following exposure of cells to classical ADAM activators. Most stimuli provoke increases in cellular Ca^2+^-levels, and in these cases, the Ca^2+^-dependent anoctamins were identified as the scramblases responsible for PS externalization. A scramblase responsible for PS exposure upon stimulation with PMA was not identified.

Xkr-proteins comprise a second major scramblase family. Xkr8 was originally identified as a scramblase that is activated by caspase-mediated cleavage during apoptosis [10,41]. However, caspase-independent activity has also been described. With regard to the latter, overexpression of Xkr8 in murine myoblasts was found to induce the formation of large myotubes during early differentiation [42]. This phenotype was not related to caspase-dependent cleavage of Xkr8 because treatment with the pan-caspase inhibitor ZVAD did not affect myotube formation. The mechanism of Xkr8 activation was not determined.

Recently, Sakuragi et al. identified three sites in the C-terminal region of murine Xkr8 whose kinase-mediated phosphorylation led to constitutive PS-exposure [32]. This region is conserved in mammalian Xkr8. The protein kinase C activator PMA represents the commonly used classical activator of ADAM17-mediated shedding and, in our previous study, was shown to vigorously provoke PS-externalization. Furthermore, we discerned that PMA-stimulation led to increased release of the ADAM17 substrates EREG and TGF-alpha in Xkr8-overexpressing HEK cells. The inhibitory effect of the specific monoclonal antibody D1 and the analysis of ADAM10/17 double-deficient HEKs identified ADAM17 as the key sheddase involved. Via its phosphorylation-prone activation, Xkr8 emerges as a potential candidate responsible for the PMA-induced triggering of ADAM-sheddase function.

At this juncture, it is pertinent to note that PS exposure is regulated not only by scramblases but also by flippases. PMA can promote endocytosis and down-regulation of certain flippases, and this could obviously augment PS-exposure in the cells [43].

The induced shedding activity of ADAM17 is influenced by a variety of mechanisms. Of particular importance are the inactive rhomboid proteins (iRhoms). iRhom2, which mediates the trafficking of ADAM17 through the secretory pathway to the cell surface, is involved in the rapid activation of ADAM17-mediated substrate release [44,45,46]. The activation mechanism involves phosphorylation of the N-terminal cytoplasmic domain of iRhom2, which leads to dissociation from ADAM17, thus promoting the increased release of substrates. We propose that both mechanisms, namely activation by iRhom2 phosphorylation to release ADAM17 and PS externalization to direct the protease to its substrate, act together in the rapid kinase-dependent ADAM17 activation pathway. Whether iRhoms might play a role in caspase-dependent ADAM17 activation remains to be elucidated. 

In this study, we also observed enhanced constitutive PS scrambling upon Xkr8 overexpression in HEK cells. While ADAM17-expression remained unaltered, there was a very enhanced release of the ADAM17 substrates EREG and TGF-alpha. The application of PS-head group OPS confirmed the functional connection to PS exposure. The observed increase in shedding activity was not calcium- or caspase-dependent. The data suggest that constitutive human Xkr8 activity may be upheld via additional mechanisms that remain to be identified.

Xkr8 is the central scramblase responsible for PS externalization in cells undergoing apoptosis. While it is known that ADAM function is increased in apoptotic cells, this event has not been connected to Xkr8 activation to date. It is pertinent to note, however, that Raji lymphoma cells lack Xkr8 because the expression is repressed due to CpG methylation near the transcription start site of the *Xkr8* gene [10]. We have reported that these cells undergo apoptosis without PS exposure and upregulation of sheddase activity [28]. The major thrust of our investigation built on this previous finding and directly addressed the possible links between the caspase-mediated activation of Xkr8 and the ADAM-signaling platform. 

HEK293T cells endogenously have low levels of ADAM-substrates, and they are readily amenable to transfection. Experiments could therefore be conducted with cells co-transfected with scramblase and the AP-tagged EGFR-ligands TGF-alpha and EREG. Apoptosis induction with kTRAIL led to markedly enhanced PS exposure in cells overexpressing Xkr8, and this was accompanied by the augmented release of both ADAM17 substrates. That this might have derived from increases in cell-surface levels of the substrates cannot be entirely excluded. However, augmented shedding effects were dose-dependently reduced in the presence of OPS, attesting to the specific involvement of PS in upregulating sheddase function. In contrast to previous results obtained in anoactamin-overexpressing calcium-activated cells, no shift in substrate specificity was noted in cells that overexpressed Xkr8. This substrate fidelity possibly reflects the specific importance of ADAM10 and ADAM17 in their function as rescue sheddases in apoptosis.

Complementary gene-silencing experiments were conducted with Caco-2 tumor cells that express high constitutive levels of Xkr8 and ADAM17 substrates. Transfection with Xkr8 siRNA led to reduced PS exposure and reduced release of the ADAM17 substrate EREG upon induction of apoptosis. There were no obvious changes in the expression of ADAM17. EREG is a growth factor that has been reported to counter the apoptosis cascade [18,38]. In our experiments, a decrease in EREG release was indeed observed to be accompanied by enhanced apoptotic cell death. The fact that inhibition of ADAM17 reduced cell viability to a comparable extent as Xkr8 siRNA is in line with the notion that one or several growth factors liberated by ADAMs may increase cellular resilience to apoptosis. The observations made in this study are complemented by the fact that ADAM17 activation also leads to the shedding of TNF-receptor 1, which might additionally reduce the sensitivity of cells towards apoptotic stimuli of the immune system [47,48,49,50]. Since siRNA experiments have a potential risk of off-target effects, further experiments with additional siRNAs or other knockdown methods would be helpful to confirm our results. 

It is of distinct interest that Xkr8-knockdown in HEK-cells that very weakly express this scramblase produced no effects similar to those observed in Caco-2 cells. This leads us to surmise that other members of the Xkr-family assume the apoptosis-related scramblase function in cells with endogenously low levels of Xkr8. Xkr4 and Xkr9 are already known to be activated by caspases [11]. Future work will test the possibility that, depending on cell type, different members of the SKr-family may replace each other to fulfill their uniform function.

In sum, we hypothesize that activation of caspase-activatable scramblases may play a net protective role in cells attacked by cytotoxic lymphocytes and NK-cells and propose that ADAM-activation is thereby of significance. This ties in perfectly with emerging concepts on the multifaceted roles of externalized PS in infections and cancer. PS exposure on tumor cells is thought to be immunosuppressive and to augment tumor growth and metastasis [51], and targeting PS is currently being discussed as a novel strategy for cancer therapy [36,52]. Silencing Xkr8 has been reported to provide anti-tumor effects in mice [39]. In line with the present results in Caco-2 cells, co-delivery of Xkr8 siRNA and a chemotherapeutic agent led to drastic inhibition of tumor growth in colon and pancreatic cancer models [36]. 

The following model is advanced to integrate ADAMs into the overall concept (Figure 5C). Extrinsic induction of apoptosis initiates the caspase cascade and activation of Xkr8-mediated PS externalization. PS-exposure provides an “eat me” signal but also concomitantly triggers “rescue” events via ADAM-activation. The release of growth factors will thereby directly counter apoptosis. Shedding of TNF-receptors might further reduce the susceptibility of tumor and virus-infected cells to immune attack. 

## 5. Conclusions

Apoptosis induction and PS externalization resulting from activation of human scramblase Xkr8 lead to upregulation of ADAM17 sheddase function. This subserves rescuing functions in cells targeted for apoptosis. Targeting Xkr8 and related scramblase expression in malignant cells might prove beneficial through the reduction of ADAM-mediated release of cell survival signals.

## Data Availability

Not applicable.

## Supplementary Materials

The following supporting information can be downloaded at: https://www.mdpi.com/article/10.3390/membranes13080720/s1, Figure S1: Expression and cell surface localization of Xkr8 upon overexpression in HEK cells. Figure S2: ADAM17 protein expression upon Xkr8 overexpression or downregulation. Figure S3: TGF-alpha-AP and EREG-AP release in ADAM10/ADAM17 double-deficient HEK cells. Figure S4: Induction of apoptosis (Caspase-3 activation) in Caco-2 cells. Figure S5: ATP levels of mock-treated Caco-2 cells. Figure S6: Downregulation of Xkr8 in HEK cells does not change kTRAIL-induced PS externalization nor substrate shedding.
