# Supplementary material for: Breakdown of Phospholipid Asymmetry Triggers ADAM17-Mediated Rescue Events in Cells Undergoing Apoptosis
Source: Membranes (Basel). 2023 Aug 5;13(8):720. doi: 10.3390/membranes13080720 (PMC10456294; doi:10.3390/membranes13080720)
Supplement: Supplementary file 1 [file membranes-13-00720-s001.zip › membranes-2478074-supplementary.pdf]

## Supplementary Figure S1

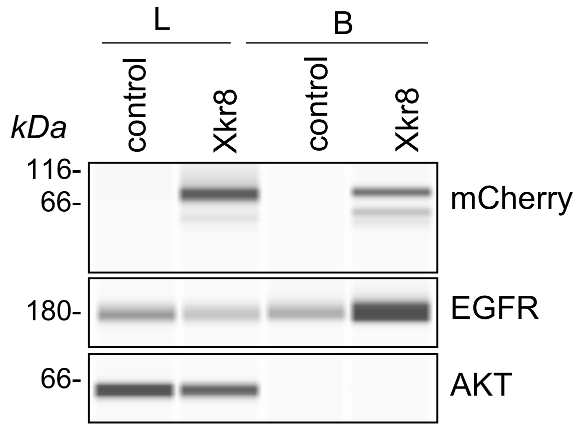


**Suppl. Figure 1**: Expression and cell surface localization of Xkr8. HEK293T cells were transfected with Xkr8-mcherry and after 48 h biotinylation experiments were performed. Transfected HEK293T cells were biotinylated and whole lysates (L) were compared with biotinylated proteins (B) by Automated Western. EGFR detection was used as positive control for cell surface proteins and AKT was used as control to exclude the detection of cytosolic proteins. Representative data of three independent experiments are shown. The intensity of the EGFR bands of Xkr8 transfected cells showed some variations in different experiments without a clear tendency indicating no causal effect.

## Supplementary Figure S2

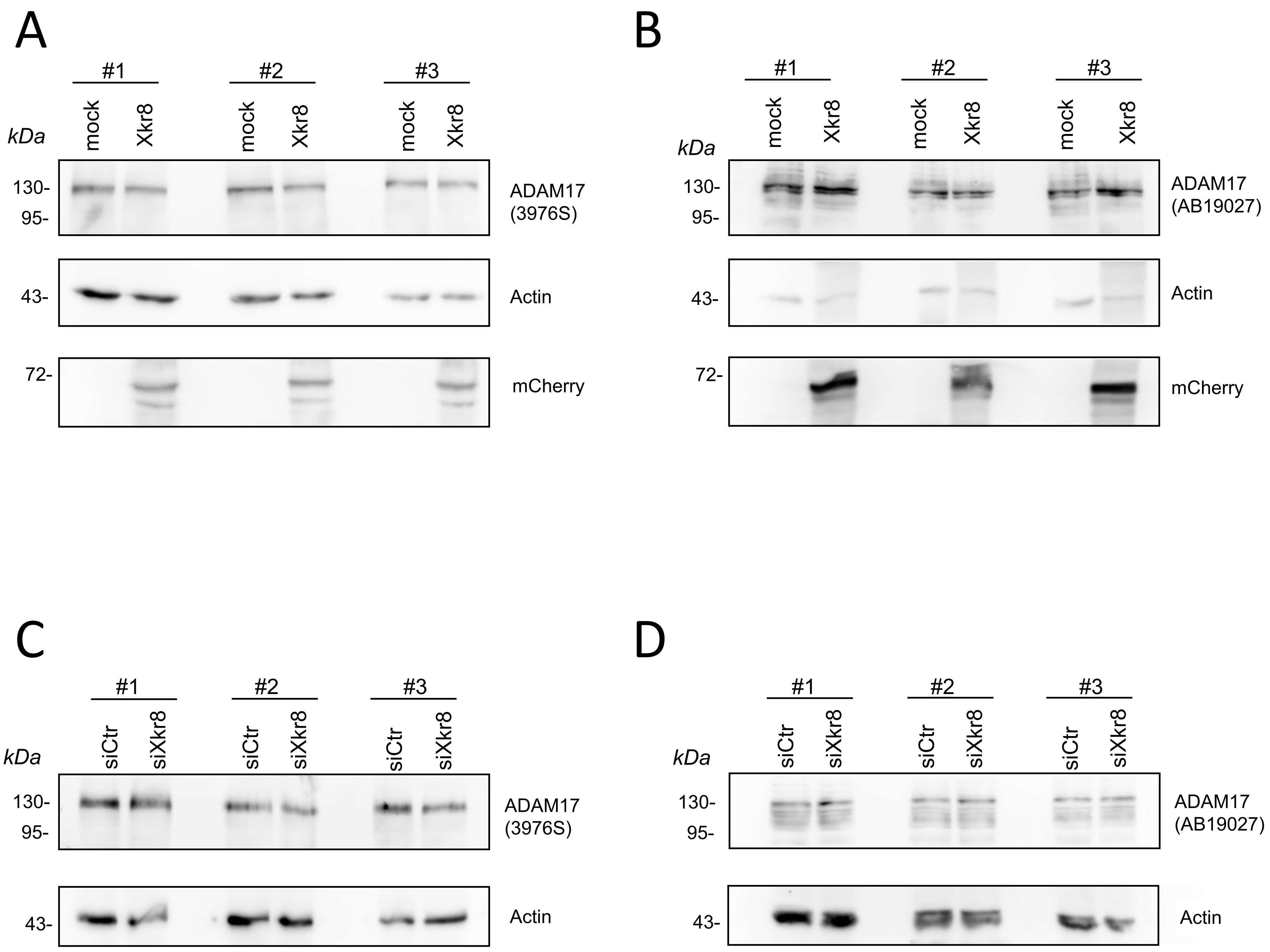


**Suppl. Figure 2**: Modulation of XKR8 expression does not lead to obvious differences in ADAM17 protein expression in HEK293T cells or Caco-2 cells. (**A/B**) HEK293T cells were mock transfected or transfected with Xkr8-mCherry plasmid and subjected to immunoblot analysis with two different anti-ADAM17 antibodies (**A**, Cell Signaling Technology, #3976S; **B**, Merck Millipore #AB19027). Cell lysates of three independent experiments were analysed (#1-3). Actin staining was used as loading control. Anti-mCherry staining confirmed successful transfection. (**C/D**) Caco-2 cells were either transfected with control siRNA (siCtr) or with Xkr8 siRNA (siXkr8). After 72 h, cells were analysed for ADAM17 protein expression via immunoblot with two different anti-ADAM17 antibodies (**C**, Cell Signaling Technology, #3976S; **D,** Merck Millipore #AB19027). Cell lysates of three independent experiments were analysed (#1-3). Actin staining was used as loading control. Successulf reduction of Xkr8 expression was confirmed via qRT-PCR (see Figure 4B).

**Supplementary Figure S3**

ADAM10/ADAM17-double-deficient HEK293 cells

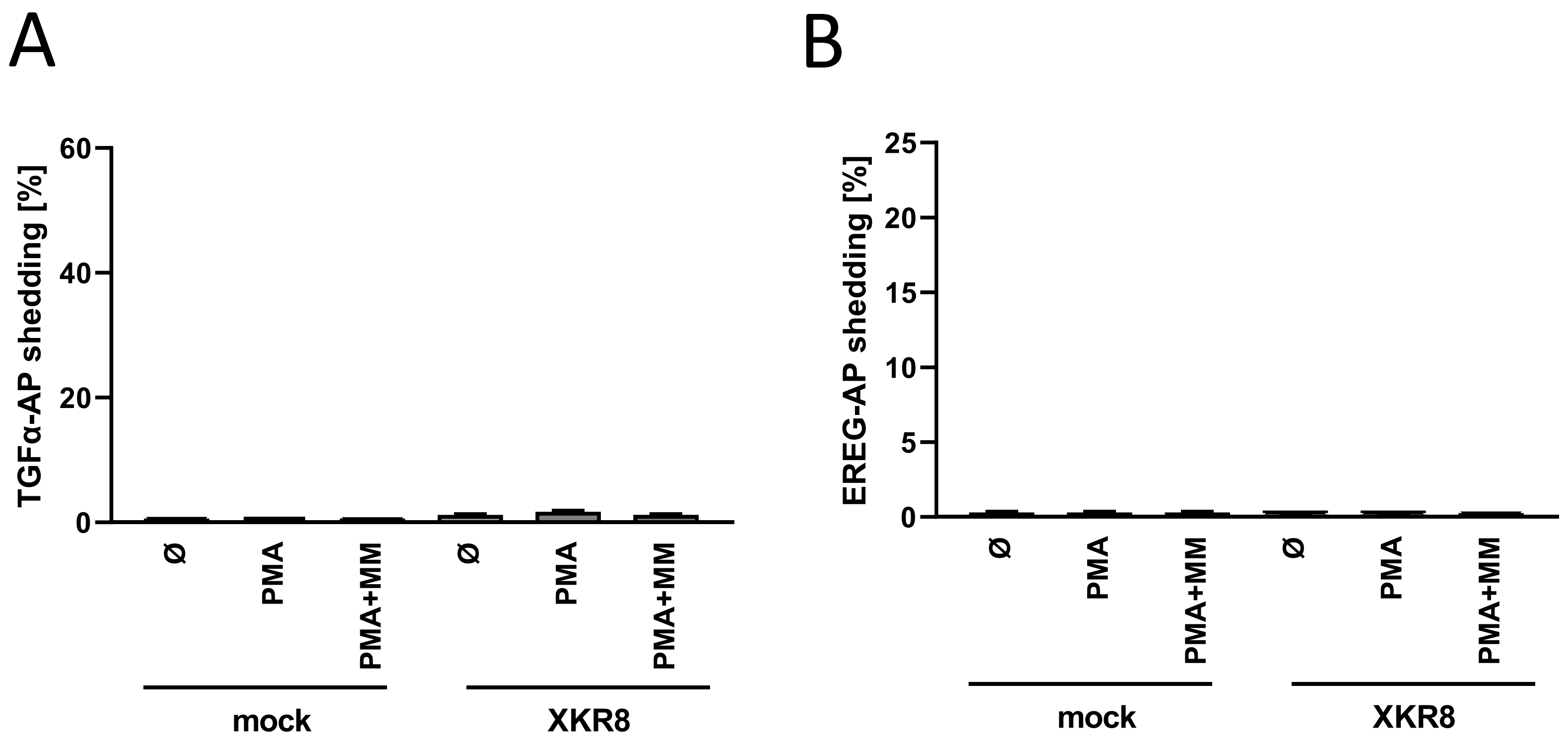


**Suppl. Figure 3**. ADAM10/ADAM17 double-deficient HEK293 cells do not shed substantial levels of TGF-alpha (**A**) or EREG (**B**) upon mock or Xkr8 transfection. ADAM10/ADAM17 double-deficient HEK cells were co-transfected with mock vector or Xkr8 and (**A**) AP-tagged TGF-alpha or (**B**) EREG, respectively. Cells were stimulated with PMA (100 ng/ml) for 30 min and analyzed for substrate shedding in the presence or absence of broad-spectrum metalloprotease inhibitor marimastat (MM, 10 µM). n = 4; ±SEM.

**Supplementary Figure S4**

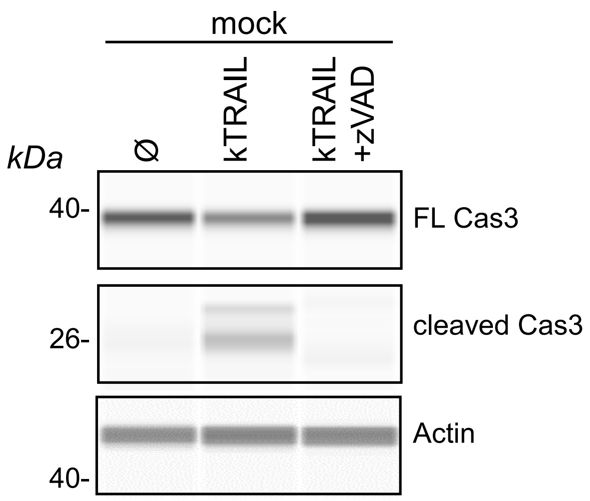


**Suppl. Figure 4**: Induction of apoptosis in Caco-2 cells. Cells were either treated with CHX (5 µg/ml) only as mock control (Ø) or treated with kTRAIL (100 ng/ml) and CHX in the absence or presence of zVAD (50 µM) over night. Cells were analyzed for expression of full length caspase-3 (FL Cas3) and activated cleaved caspase-3 (cleaved Cas3) by Automated Western. Actin was used as loading control. Representative data of three independent experiments are shown.

# Supplementary Figure S5

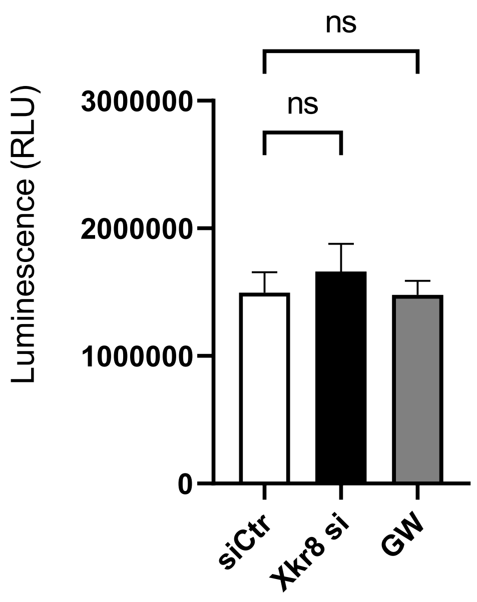


**Suppl. Figure 5**: No significant differences in ATP levels of mock-treated Caco-2 cells. Caco-2 cells were either transfected with control siRNA (siCtr) or with Xkr8 siRNA (Xkr8 si). Mock-transfected cells were additionally incubated in the presence of ADAM10/ADAM17 inhibitor GW (3 µM). Luminescence was measured as an indicator of cell viability (ATP) using CellTiter-Glo Assay (Promega) according to the manufacturer's instructions ($n = 3$; $*p < 0.05$; ±SEM). Ns indicates no significant difference. Data were analyzed by one-way analysis of variance and Holm-Sídák multiple comparison post hoc test.

## Supplementary Figure S6

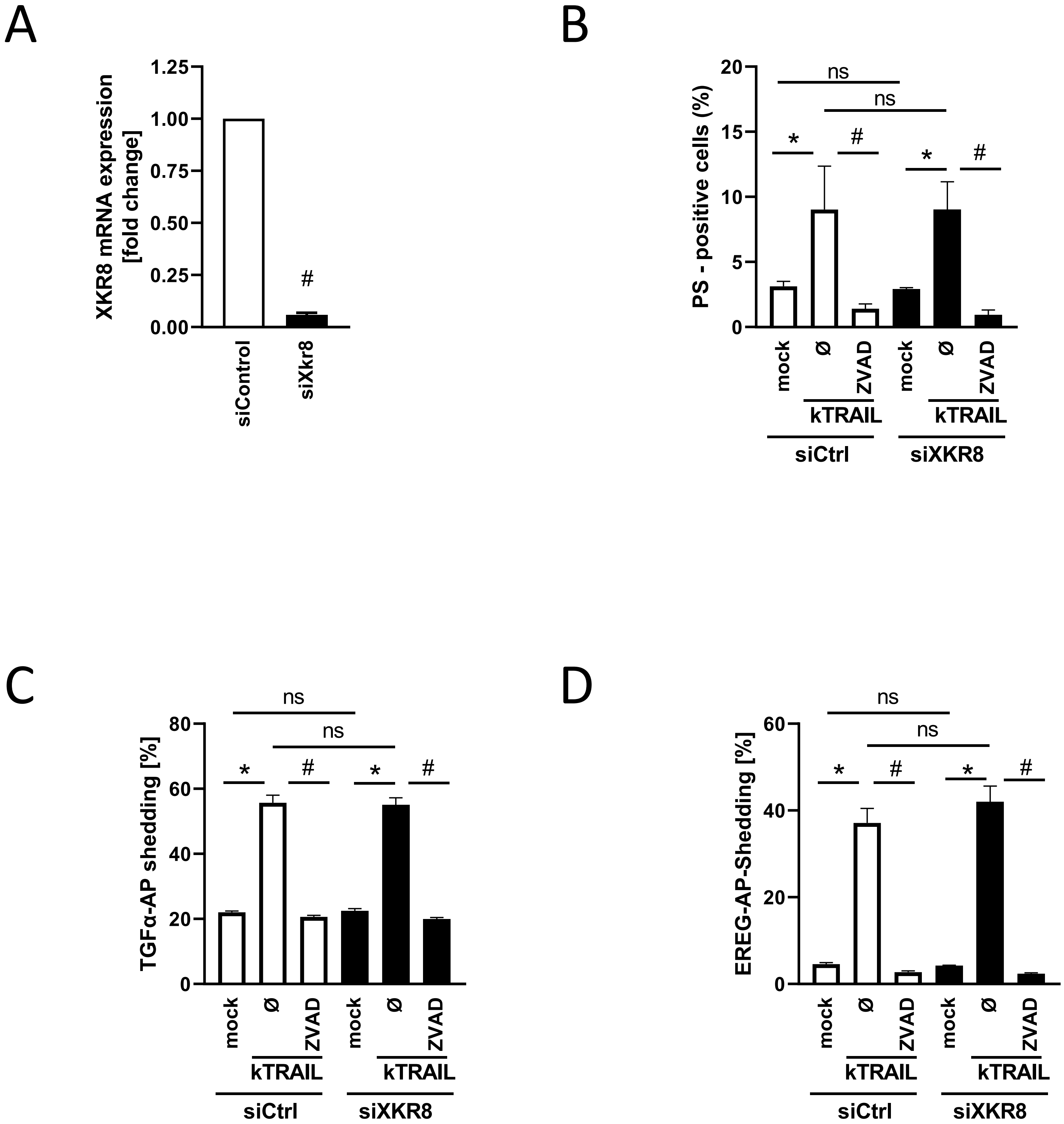


**Suppl. Figure 6**: Decreased expression of *Xkr8* in HEK cells does not lead to decreased kTRAIL-induced PS exposure nor substrate shedding. (**A**) HEK cells were either transfected with control siRNA or with Xkr8 siRNA. After 72 h, cells were analyzed for *Xkr8* expression by qRT-PCR. #significant decrease ($n = 3$; # $p < 0.05$; ±SEM; unpaired t-test). (**B**) Caspase-dependent PS exposure is not significantly affected in Xkr8 siRNA transfected HEK cells. Mock transfected and Xkr8 siRNA transfected cells were stimulated with CHX (5 µg/ml) alone (mock) or with kTRAIL (50 ng/ml) and CHX in the absence or presence of ZVAD (50 µM), stained for 15 min with Annexin V-488 and 7-AAD for exclusion of dead cells and analysed via FACS. Quantifaction of viable PS-positive cells of three independent experiments is shown ($n = 3$; */# $p < 0.05$; ±SEM). Ns = no significant difference. Data were analyzed by one-way analysis of variance and Holm-Sídák multiple comparison post hoc test. (**C/D**) HEK293T cells were transfected control siRNA or with Xkr8 siRNA and 24 h later with AP-tagged TGF-alpha (**C**) or epiregulin (**D**), respectively. 48 h after siRNA transfection, cells were stimulated CHX (5 µg/ml) alone (mock) or with kTRAIL (50 ng/ml) and CHX (5 µg/ml) overnight in the presence of absence of caspase inhibitor ZVAD (50 µM). Constitutive and apoptosis-induced shedding was not significantly changed upon overexpression of Xkr8. *Significant increase compared with indicated control, # significant decrease compared with respective kTRAIL-treated cells. Ns = no significant difference. $n = 3$; */# $p < 0.05$; ± SEM. Data were analyzed by one-way analysis of variance and Holm-Sídák multiple comparison post hoc test.
